# Familial aggregation analysis of gene expressions

**DOI:** 10.1186/1753-6561-1-s1-s49

**Published:** 2007-12-18

**Authors:** Shao-Qi Rao, Liang-De Xu, Guang-Mei Zhang, Xia Li, Lin Li, Gong-Qing Shen, Yang Jiang, Yue-Ying Yang, Bin-Sheng Gong, Wei Jiang, Fan Zhang, Yun Xiao, Qing K Wang

**Affiliations:** 1 Department of Bioinformatics, Harbin Medical University, Harbin 150086, People's Republic of China; 2 Departments of Molecular Cardiology and Cardiovascular Medicine, The Cleveland Clinic Foundation, 9500 Euclid Avenue, Cleveland, Ohio 44195, USA; 3 Biomedical Engineering Institute, Capital University of Medical Sciences, Beijing 100054, People's Republic of China; 4 The First Clinical College, Harbin Medical University, Harbin 150081, People's Republic of China; 5 Department of Computer Science, Harbin Institute of Technology, Harbin 150080, People's Republic of China

## Abstract

Traditional studies of familial aggregation are aimed at defining the genetic (and non-genetic) causes of a disease from physiological or clinical traits. However, there has been little attempt to use genome-wide gene expressions, the direct phenotypic measures of genes, as the traits to investigate several extended issues regarding the distributions of familially aggregated genes on chromosomes or in functions. In this study we conducted a genome-wide familial aggregation analysis by using the in vitro cell gene expressions of 3300 human autosome genes (Problem 1 data provided to Genetic Analysis Workshop 15) in order to answer three basic genetics questions. First, we investigated how gene expressions aggregate among different types (degrees) of relative pairs. Second, we conducted a bioinformatics analysis of highly familially aggregated genes to see how they are distributed on chromosomes. Third, we performed a gene ontology enrichment test of familially aggregated genes to find evidence to support their functional consensus. The results indicated that 1) gene expressions did aggregate in families, especially between sibs. Of 3300 human genes analyzed, there were a total of 1105 genes with one or more significant (empirical *p *< 0.05) familial correlation; 2) there were several genomic hot spots where highly familially aggregated genes (e.g., the chromosome 6 HLA genes cluster) were clustered; 3) as we expected, gene ontology enrichment tests revealed that the 1105 genes were aggregating not only in families but also in functional categories.

## Background

Familial aggregation is the more frequent occurrence of a trait in members of a family than among non-related individuals. Thus, it is a common analysis method to determine the genetic contribution to a complex human disease. Technically, this type of analysis is a more detailed version of the mixed linear model approach in that each type of relative pairs is estimated separately instead of modeling them as a function of a few parameters in a single covariance matrix. Historically, familial aggregation analysis has been the most popular method for determining genetic causes of disease. This method, in essence, is to estimate the correlations between various biological relatives and then similarly assume that they can be parsimoniously explained by an additive genetic contribution and a common household contribution, but without making all the other assumptions of the mixed linear model. Although familial aggregation has been well studied for many diseases [1], genome-wide gene expressions typically have not been used as the traits. Problem 1 data for Genetic Analysis Workshop 15 (GAW15), initially used for mapping expression quantitative trait loci [2], provided expression levels of 3554 genes in lymphoblastoid cells for 14 three-generation CEPH (Centre d'Etude du Polymorphisme Humain) Utah families. Because of their inborn nature, expression of these genes might be less affected by a list of environmental factors for complex human diseases. Therefore, the specific aims of the present study were to answer three genetics questions: 1) how gene expressions aggregate among different types (degrees) of relative pairs; 2) how they are distributed on chromosomes; and 3) what functional implications they have.

## Methods

### Description of the data set

Expression levels of genes in lymphoblastoid cells of each individual of 14 three-generation CEPH Utah families (~8 offspring per sibship, ~14 individuals per family, total of 194 individuals) were provided for GAW15 Problem 1. For 3554 of the 8500 genes tested, Morley et al. [2] found greater variation among individuals than between replicate determinations on the same individual. We further reduced the above number of genes to 3300 by deleting the genes having uncertain chromosome locations or situated on chromosomes X and Y.

### Calculating familial correlations

S.A.G.E FCOR [3] can be used to calculate familial correlations for a variety of biological relative types. Here, this module was used to calculate familial correlation (*R*) for 15 relative types: father-son (FS), mother-son (MS), father-daughter (FD), mother-daughter (MD), brother-brother (BB), sister-brother (SB), sister-sister (SS), grandfather-father-grandson (FFS), grandmother-father-grandson (MFS), grandfather-mother-grandson (FMS), grandmother-mother-grandson (MMS), grandfather-father-granddaughter (FFD), grandmother-father-granddaughter (MFD), grandfather-mother-granddaughter (FMD) and grandmother-mother-granddaughter (MMD). As reported by S.A.G.E. PEDINFO [3], the CEPH Utah families provided 220 parent-offspring pairs, 378 sibling pairs, and 440 extended relative pairs. To test the statistical significance of a correlation estimate and to correct for multiple tests for 15 relative types, we also performed 100,000 permutations on the 3300 × 15 (genes × number of relative types) matrix. The empirical thresholds are *R *= 0.4609 and *R *= 0.6532, respectively, for the significant levels of 0.05 and 0.01.

### Gene ontology (GO) enrichment analysis of familially aggregated genes

To see if the genes significantly aggregating in families are also aggregating in functional categories, we performed a gene ontology (GO) enrichment test. Suppose that a total of *N *(3300) genes (set A) for the analyzed data are annotated in GO in which a set of *M *(1105 found in this study) genes (set B) are significantly familially aggregated. For a given GO category, a gene is either in the category or not in the category. Suppose further that *n *genes out of set A and *m *genes out of set B are in the category. If the *m *significantly aggregated genes are effectively a random sample uniformly selected from set B, the expected value of *m *is (*n*/*N*)*M*. Because a gene can be selected only once, this is sampling without replacement and can therefore be appropriately modeled by a hypergeometric distribution [4]. The probability of observing at least *m *significantly familially aggregated genes in the GO category of *n *genes can be computed as follows:

$$p=1-\sum_{i=0}^{m-1}\frac{\left(\begin{array}{l} M\\ i \end{array}\right)\left(\begin{array}{l} N-M\\ n-i \end{array}\right)}{\left(\begin{array}{l} N\\ n \end{array}\right)}.$$

The *p*-value calculated above corresponds to a one-sided test and a smaller *p*-value relates to a higher likelihood of a GO category's enrichment with genes that aggregate significantly in families. In this study, to avoid the possible loss of the true positives, we identified significant GO categories on the basis of the criterion of nominal significance of *p *≤ 0.01. Therefore, the *p*-value quoted should be considered as a heuristic measure, useful as an indicator that roughly rates the relative enrichment of significantly familially aggregated genes for each GO category.

## Results

### How do gene expressions aggregate among different types of relative pairs?

**Figure 1:**
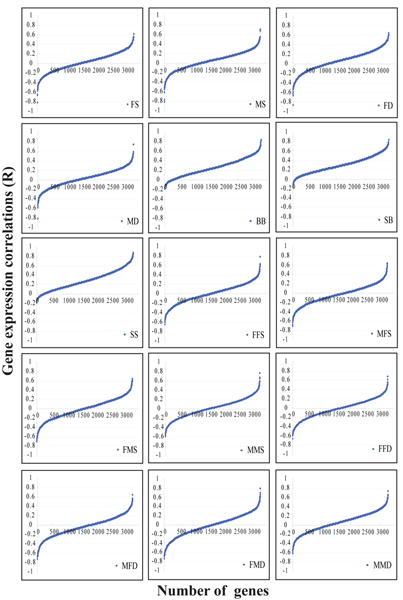
**Gene expression correlation distributions in different types of relative pairs**. Each panel is for a type of relative pairs. The transverse axis stands for the ordered number of the 3300 studied genes and the longitudinal one denotes the expression correlations.

Of 3300 genes evaluated, we found 1105 genes having one or more significant (empirical *p *≤ 0.05) familial correlation, and 212 genes having one or more highly significant (*p *≤ 0.01) familial correlation. Table 1 shows the distributions of correlation estimates for the 3300 genes per the relative types. Sibling correlations were the highest, as expected by quantitative genetics theory, possibly due to a larger shared non-genetic component. Further examination of the pool of significant sibling correlations, revealed that about half of them (291 genes) were shared by the three types of sibling pairs, thus likely to be gender-independent. Also, the higher correlations between the more closely related pairs were in agreement with quantitative genetics theory [5]. It is interesting to observe that at the level of 0.01, the number of significant correlation estimates was dramatically reduced for all the relative types. We plotted the distributions of familial correlations for all 3300 genes per relative type. The distributions of correlations for the 15 types of relative pairs (Fig. 1) show that 1) the correlation estimates for brother-brother, sister-brother, and sister-sister were skewed to positive with few negative estimates; 2) much larger proportions of the estimates for the remaining 12 types were negative.

**Table 1:** Numbers of significant familial correlations (*R*)

Type	Observed number of pairs	*R *> 0.4609	*R *> 0.6532
		
		(*P *< 0.05)	(*P *< 0.01)
Father-son	57	36	0
Mother-son	57	32	2
Father-daughter	53	78	0
Mother-daughter	53	20	1
Brother-brother	105	532	48
Sister-brother	179	676	104
Sister-sister	94	688	161
Grandfather-father-grandson	57	29	1
Grandmother-father-grandson	57	19	0
Grandfather-mother-grandson	57	34	0
Grandmother-mother-grandson	57	37	3
Grandfather-father-granddaughter	53	16	1
Grandmother-father-granddaughter	53	19	0
Grandfather-mother-granddaughter	53	48	2
Grandmother-mother-granddaughter	53	69	3

### How are familial correlations distributed (aggregated) over chromosomes?

**Figure 2:**
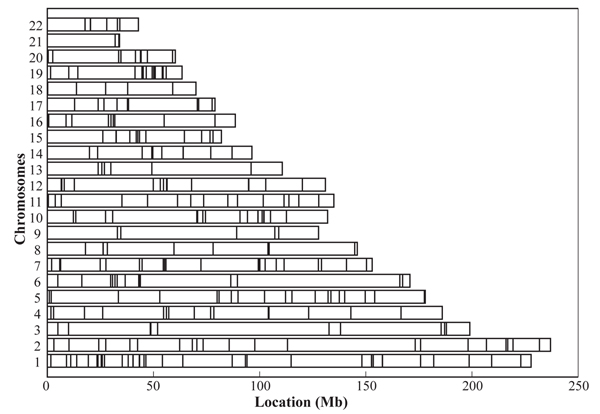
**Distributions of familial (brother-brother) correlations over chromosomes**. The horizontal bar is the relative length of chromosomes, and the vertical lines on chromosomes are used to indicate the approximate positions of the genes with significant BB correlations.

To answer the question, Figure 2 was made to visualize the aggregation of the genes with significant brother-brother correlations (*p *≤ 0.05) on autosomes according to the relative physical distances of these genes. It can be easily seen that the genes are not uniformly distributed on the chromosomes and form at least one highly aggregated region on each chromosome. It is noteworthy to look at a highly aggregated 5-Mb band (29–34 Mb) on chromosome 6, which contains six genes, three of which have direct relevance to immunology. Genes *HLA-DOA *(Gene_ID 3111, 33082315–33085367) and *HLA-F *(Gene_ID 3134, 29801690–29802280) are important known immunological genes. *TAP2 *(GENE_ID 6891, 32904275–32914499) is a neighbor of the HLA cluster, which encodes a protein participating in an antigen representation process. *VARS *(GENE_ID 7407, 31853277–31871489) is an aminoacyl-tRNA synthetase. *CLIC1 *(GENE_ID 1192, 31806342–31812292) encodes chloride intracellular channel 1. A protein encoded by *PPP1R10 *(GENE_ID 5514, 30676161–30692987) has an inhibitory effect on protein phosphatase-1.

### How are significantly familially aggregated genes aggregating in function categories?

We put all genes onto gene ontology (GO) [6] to get the categories. Then we selected the GO categories that contained at least five genes. Next, the hypergeometric test was applied to obtain a *p*-value of each studied category for its enrichment with significantly familially aggregated genes (i.e., from set B of 1105 genes). We found that more than one-third of the studied categories (36 out of 100 molecular function categories, 49 out of 119 biology process categories) were significantly enriched. However, taking into account the 229 categories evaluated, and using the very conservative Bonferroni correction, only six molecular function categories (GO categories one to six shown in Table 2) and four biological process categories (data not shown) remain significant. Table 2 lists the highly significantly (nominal *p *≤ 0.01) enriched GO molecular function categories. It is interesting to note that the majority of the enriched GO categories relate to molecular binding, transcription factor, ligase activity, and receptor activity.

**Table 2:** A list of GO molecular function categories highly significantly (*p *≤ 0.01) enriched with genes of high familial aggregations

GO ID	GO Description	*n*^a^	*m*^b^	*p*
GO:0000166	nucleotide binding	468	202	6.15 × 10^-7^
GO:0003735	structural constituent of ribosome	54	5	2.5 × 10^-5^
GO:0003723	RNA binding	177	83	4.64 × 10^-5^
GO:0004872	receptor activity	174	37	9.95 × 10^-5^
GO:0005509	calcium ion binding	150	31	0.000154
GO:0051082	unfolded protein binding	66	36	0.000187
GO:0005524	ATP binding	377	152	0.000578
GO:0005515	protein binding	880	264	0.001239
GO:0004004	ATP-dependent RNA helicase activity	6	6	0.001397
GO:0016874	ligase activity	73	35	0.003392
GO:0003779	actin binding	57	10	0.003461
GO:0003700	transcription factor activity	232	63	0.005974
GO:0004842	ubiquitin-protein ligase activity	31	17	0.007186
GO:0003676	nucleic acid binding	80	36	0.008773
GO:0005506	iron ion binding	57	27	0.009803

^a^*n*, Number of genes contained in a category identified and counted by using set A (a total of 3300 genes).

^b^*m*, Number of (highly familially aggregated) genes contained in the category, counted using set B (a total of 1105 genes).

## Discussion

To the best of our knowledge, this study is the first attempt to relate the familial aggregation patterns of genes with their genomic locations and their functionalities. Familial aggregation analysis of a large number of genes using different relative types suggests that some non-Mendelian genetic factors or environment factors may affect these gene expressions too, such as age-dependent genetic imprinting [7] or antagonistic environments for family members in different generations, possibly leading to biased estimates of some familial correlations. Regarding the use of different relative types for estimating additive genetic effects in gene expressions, it appears that no single relative type stands out as the best for all scenarios. The results acquired from this analysis of genome-wide gene expression traits raise a paradoxical challenge regarding the use of familial aggregation analysis to determine the genetic contribution to a quantitative trait. On one hand, the use of sibling pairs is favored because it is unlikely to produce a negative estimate of heritability, but tends to overestimate it because of the larger shared non-genetic components and dominance components. On the other hand, the use of other relative pairs is unlikely to overestimate heritability, but can be problematic if some factor(s) (e.g., antagonistic environments) causes the familial individuals between different generations to be environmentally negatively correlated.

Further bioinformatics analysis of familial aggregated genes suggests some consistencies between familial aggregations and chromosomal aggregations and functional aggregations. However, we feel that these exploratory results warrant further investigation because of the limited sample size used in the study. In addition, traditional quantitative genetics approaches, which assume a polygenic basis for the studied traits and normal distribution of the underlying genetic effects, might not be the most appropriate to analyze the expression phenotypes whose genetic models could be monogenic or oligogenic. Although the familial aggregation analysis approach as implemented in S.A.G.E. is robust to non-normality of traits, further study is needed regarding our method's behaviors and properties when applied to traits having a genetic basis quite deviated from what is expected for truly quantitative traits.

## Conclusion

Most of our results from the genetic epidemiological analysis were consistent with quantitative genetics theory. Further bioinformatics analysis revealed that familially aggregated genes tended to aggregate on some genomic regions and to enrich their functional categories.

## Competing interests

The author(s) declare that they have no competing interests.
